# Editorial: Innovative Strategies From Synthetic Biology and Bacterial Pathways to Master Biochemical Environmental Challenges

**DOI:** 10.3389/fbioe.2021.828632

**Published:** 2022-01-11

**Authors:** Frank Eisenhaber, Juilee Thakar, Alicia Ponte-Sucre, Thomas Dandekar

**Affiliations:** ^1^ Bioinformatics Institute (BII), Agency for Science, Technology and Research (A*STAR), Singapore, Singapore; ^2^ Genome Institute of Singapore (GIS), Agency for Science, Technology and Research (A*STAR), Singapore, Singapore; ^3^ School of Biological Sciences, Nanyang Technological University (NTU), Singapore, Singapore; ^4^ Department of Microbiology and Immunology, Department of Biomedical Genetics and Department of Biostatistics and Computational Biology, University of Rochester Medical Center, Rochester, NY, United States; ^5^ Laboratorio de Fisiología Molecular, Instituto de Medicina Experimental, Escuela Luis Razetti, Medical Mission Institute, Universidad Central de Venezuela, Würzburg, Germany; ^6^ Department of Bioinformatics, Functional Genomics and Systems Biology Group, Biocenter, University of Würzburg, Würzburg, Germany

**Keywords:** pathway, bacteria, anthropocene, antibiotics, protein engineering, biofuels, xenobiotics, climate plants

Sustainability is increasingly becoming an important factor of this century. The scale of human activities on this Earth has reached planetary dimensions; affecting global homeostasis in an existence-threatening manner–be it pollution of oceans and atmosphere, impoverishment of the biosphere or the impending climate crisis. The reasons for affecting global homeostasis can be traced down to 1) the increase in the world population including increase by longer life span and 2) the increased consumption of goods per capita (“prosperity”). Similarly, industry produces and bombards us with a variety of new materials (“xenobiotics”). To cope with this challenge, sustainable and environmental-friendly solutions can be found in biotechnology and bioengineering. Particularly, the reservoir of bacterial pathways and their unique adaption potential can be tapped to resolve climate related problems and also for general applications. Bacteria have a surprising richness of pathways which can be leveraged to achieve several of these diverse goals. As a second option, synthetic biology has gained traction for developing ways to perturb and manipulate biochemical pathways and exploit the pathway reservoirs in all organisms. Here in our special topic we explain how both options can be turned into motion and which results and outcomes may be achieved and expected.

Chemical transformation of compounds are key elements of production processes. Traditional chemical industry becomes wasteful with increasing consumption driving higher energy and resource needs when the complexity of the reactions exceeds certain thresholds and, at the same time, the total yield required is measured in hundreds of kilograms instead of millions of tons. It is at this point where synthetic biology approaches have an essentially unlimited future. By reapplying evolutionarily optimized and, maybe, cautiously engineered biosynthetic or catabolic pathways that work at atmospheric pressure and with normal surrounding temperature for application purposes, environmentally sustainable production solutions can become not only possible but a reality. For example, how complex waste materials could be processed, and especially dispersed in nature such as plastic debris or dangerous chemical compounds, synthetic biology approaches might offer unprecedented ways out of accumulated problems (Zrimec et al., 2021).

As microbial and fungal organisms, to some extent plants, have a demonstrated large diversity of biochemical pathways and signs of continued evolution of chemical processing variety (rather in contrast to higher eukaryote animals); they are prime targets for scientific research to learn from their pathway richness how to tackle new metabolites and xenobiotics. Yet, nothing comes without investment into basic science. Many gene functions remain uncharacterized [for example in yeast (Peña-Castillo and Hughes, 2007) and in human (Sinha et al., 2018)]. Even for *E. coli*, the best studied organism on Earth, there is still a number of genes with unknown function (Mukherjee et al., 2018; Ng et al., 2018). Many microorganisms and fungi live for example at waste dump sites, in the tropical forests or in mangroves and must have developed a remarkable chemistry to survive in their environment and have until now never become a target of scientific quests. Thus, while this special issue highlights several biosynthetic gene clusters of key interest, many more potentially useful biochemical pathways and biosynthetic gene clusters are there and probably even more are yet to be discovered.

Here, we present the research topic “Innovative Strategies from Synthetic Biology and Bacterial Pathways to Master Biochemical Environmental Challenges”, a small selection of articles that, understandably, just scratches some aspects of these pathway engineering challenges. The reader should be aware that there are also numerous other efforts in industry and biotechnology, often remaining unpublished.

This collection (Figure 1) includes a paper about synthetic pathways for carbon dioxide harvesting (Osmanoglu et al.). The authors describe a synthetic biology approach that outsmarts nature since their modified *in silico* design of carbon dioxide fixation pathways in some plants may be up to five times as effective as their naturally occurring counterpart. Other papers show how to efficiently select best enzyme varieties for engineering approaches to process xenobiotics or produce new substances from huge sequence databases (Sirota et al.). These can then be applied for improved synthesis of renewable biofuels and other chemicals (Scherer et al.). Latest trends in bioremediation technologies are discussed in Dutta et al. while Bhatwa et al. tackle head-on the challenges in protein design regarding expression and industrial application. Synthetic biology approaches are the method of choice in the production of complex bioactive compounds such as antibiotics. New anti-microbial and anti-fungal compounds are needed to fight the emergence of resistant strains. Here Yele et al. suggest interesting new solutions with phenylacetamide and benzohydrazide derivatives.

**FIGURE 1:**
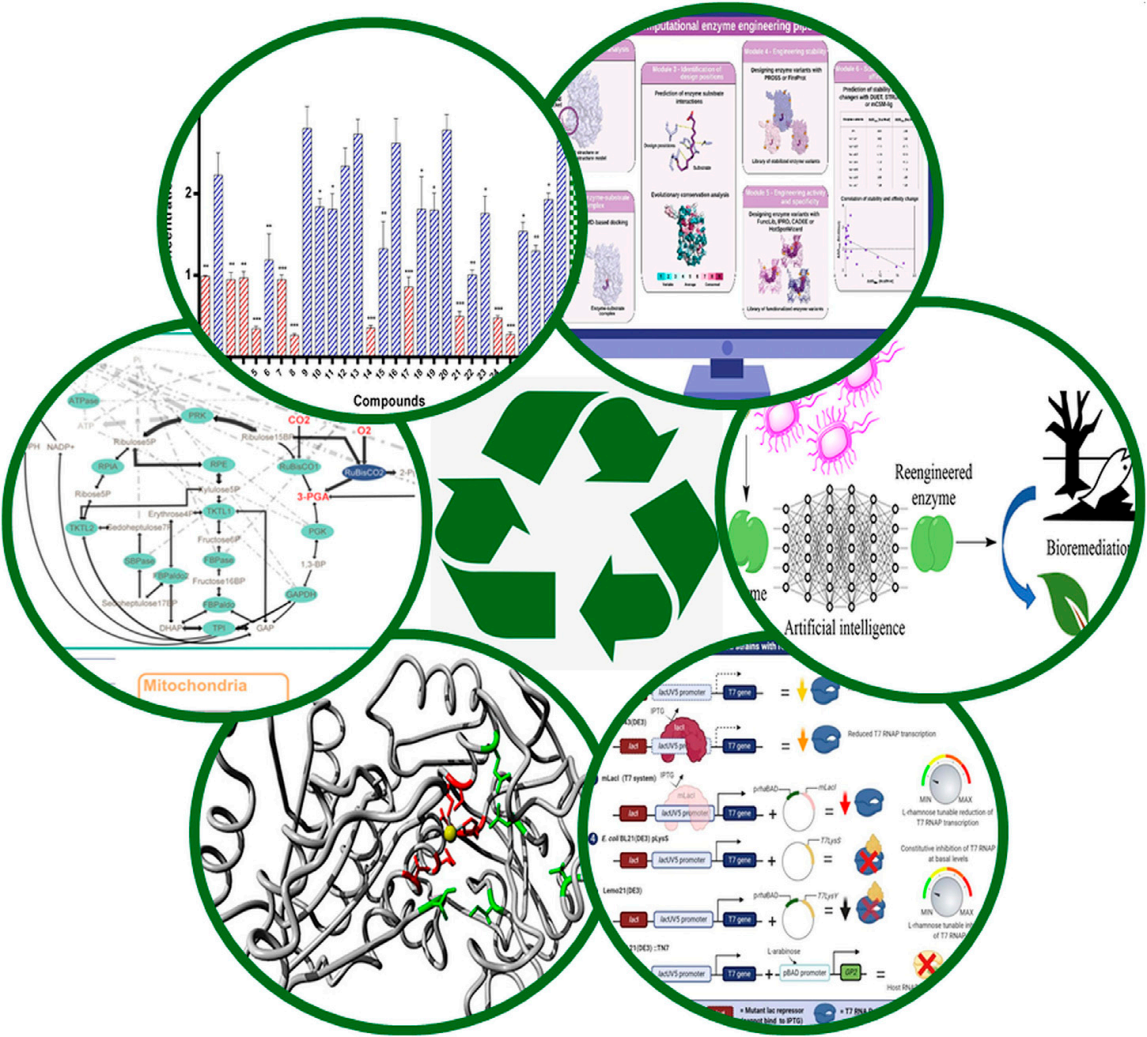
Innovative strategies from synthetic biology and bacterial pathways to master biochemical environmental challenges. Our article collection of six articles gives a multi-facetted integrated picture on the involved pathways and engineering strategies (Figure: Courtesy of Dr. Elena Bencurova).

Thus, we are convinced that synthetic biology illustrates the importance of basic science to tackle the environmental challenges humanity and the globe are facing. We also think there is an urgent need to convince the global population about the usefulness of these methodologies, that basic and applied research constitute two sides of a coin, and that in this specific case both approaches should work together to incorporate methodologies for optimal processing of wastes, xenobiotics, antibiotics and new products. The success of these aims will mean a better understanding of the need for a balanced relationship with nature. Our global civilization has become dominating in its environmental impact everywhere, this is a new era which is called the Anthropocene (Laurance, 2019). As a result, our civilization will only be sustainable and lasting in a balanced relationship with nature. This requires transformation of our current linear high productive but environmental devastating economy into a regenerative and sustainable circular economy. Bacterial pathways testify how this is done best in equilibrium with nature and they offer an overwhelming richness of solutions, strongly underestimated previously (Castelle and Banfield, 2018). Synthetic biology with protein engineering provides in addition the ability to implement such solutions fast with optimized results and for pro- and eukaryotes alike. We provide here stimulatory reading and rapid implementation suggestions covering both aspects.

## References

[B1] Castelle C. J. Banfield J. F. (2018). Major New Microbial Groups Expand Diversity and Alter Our Understanding of the Tree of Life. Cell 172, 1181–1197. 10.1016/j.cell.2018.02.016 29522741

[B2] Laurance W. F. (2019). The Anthropocene. Curr. Biol. 29 (19), R953–R954. 10.1016/j.cub.2019.07.055 31593674

[B3] Mukherjee K. Narindoshvili T. Raushel F. M. (2018). Discovery of a Kojibiose Phosphorylase in *Escherichia coli* K-12. Biochemistry 57, 2857–2867. 10.1021/acs.biochem.8b00392 29684280 PMC5953851

[B4] Ng T. W. Ip M. Chao C. Y. H. Tang J. W. Lai K. P. Fu S. C. (2018). Differential Gene Expression in *Escherichia coli* during Aerosolization from Liquid Suspension. Appl. Microbiol. Biotechnol. 102, 6257–6267. 10.1007/s00253-018-9083-5 29808326

[B5] Peña-Castillo L. Hughes T. R. (2007). Why Are There Still over 1000 Uncharacterized Yeast Genes? Genetics 176, 7–14. 10.1534/genetics.107.074468 17435240 PMC1893027

[B6] Sinha S. Eisenhaber B. Jensen L. J. Kalbuaji B. Eisenhaber F. (2018). Darkness in the Human Gene and Protein Function Space: Widely Modest or Absent Illumination by the Life Science Literature and the Trend for Fewer Protein Function Discoveries since 2000. Proteomics 18, e1800093. 10.1002/pmic.20180009 30265449 PMC6282819

[B7] Zrimec J. Kokina M. Jonasson S. Zorrilla F. Zelezniak A. (2021). Plastic-degrading Potential across the Global Microbiome Correlates with Recent Pollution Trends. mBio 12, e0215521. 10.1128/mBio.02155-21 34700384 PMC8546865

